# Ultra-fast vitrification of patient-derived circulating tumor cell lines

**DOI:** 10.1371/journal.pone.0192734

**Published:** 2018-02-23

**Authors:** Rebecca D. Sandlin, Keith H. K. Wong, Shannon N. Tessier, Anisa Swei, Lauren D. Bookstaver, Bennett E. Ahearn, Shyamala Maheswaran, Daniel A. Haber, Shannon L. Stott, Mehmet Toner

**Affiliations:** 1 BioMEMS Resource Center, Center for Engineering in Medicine & Department of Surgery, Massachusetts General Hospital, Harvard Medical School, Boston, Massachusetts, United States of America; 2 Cancer Center & Department of Surgery, Massachusetts General Hospital, Harvard Medical School, Boston, Massachusetts, United States of America; 3 Cancer Center & Department of Medicine, Massachusetts, MA General Hospital, Harvard Medical School, Boston, Massachusetts, United States of America; 4 Howard Hughes Medical Institute, Chevy Chase, Maryland, United States of America; 5 Cancer Center, Department of Medicine & BioMEMS Resource Center, Center for Engineering in Medicine, Massachusetts General Hospital, Harvard Medical School, Boston, Massachusetts, United States of America; The Ohio State University, UNITED STATES

## Abstract

Emerging technologies have enabled the isolation and characterization of rare circulating tumor cells (CTCs) from the blood of metastatic cancer patients. CTCs represent a non-invasive opportunity to gain information regarding the primary tumor and recent reports suggest CTCs have value as an indicator of disease status. CTCs are fragile and difficult to expand *in vitro*, so typically molecular characterization must be performed immediately following isolation. To ease experimental timelines and enable biobanking, cryopreservation methods are needed. However, extensive cellular heterogeneity and the rarity of CTCs complicates the optimization of cryopreservation methods based upon cell type, necessitating a standardized protocol. Here, we optimized a previously reported vitrification protocol to preserve patient-derived CTC cell lines using highly conductive silica microcapillaries to achieve ultra-fast cooling rates with low cryoprotectant concentrations. Using this vitrification protocol, five CTC cell lines were cooled to cryogenic temperatures. Thawed CTCs exhibited high cell viability and expanded under *in vitro* cell culture conditions. EpCAM biomarker expression was maintained for each CTC cell line. One CTC cell line was selected for molecular characterization, revealing that RNA integrity was maintained after storage. A qPCR panel showed no significant difference in thawed CTCs compared to fresh controls. The data presented here suggests vitrification may enable the standardization of cryopreservation methods for CTCs.

## Introduction

Circulating tumor cells (CTCs) are cells originating from the primary tumor that intravasate into blood vessels and can eventually colonize distant tissues, resulting in metastatic spread of disease, the major cause of cancer mortality. While CTCs are extremely rare cells and thought to be present at concentrations as low as 1 CTC in 10^9^ blood cells, microfluidic technologies have enabled their purification and subsequent molecular analysis from peripheral blood specimens.[1–5] Recent reports have demonstrated that CTCs may provide important details regarding the status of the primary tumor, disease prognosis and provide opportunities to improve cancer management.[6–10] To preserve this important molecular information for future clinical and research endeavors, methods that enable cryogenic storage of CTCs are needed.

Biorepositories have been established to support the collection, storage and dissemination of biological samples for clinical and research purposes. These data-rich biobanks hold collections of biological material obtained from the general population, biopsies or from the deceased and are essential for the research community and critical for the development of personalized medicine.[11, 12] Biobanking methods for CTCs could enable biomarker identification, expansion *in vitro* for drug susceptibility assays, and enable more efficient batch processing of isolates. The quality of material obtained from biobanking is critically important to the extraction of molecular information at the DNA, RNA or protein level. In the case of CTC biobanking, cell viability is important as improved methods for CTC culture and xenograft models are being reported.[13–16] However, CTCs represent unique challenges to the development of cryopreservation protocols due to their extreme rarity and heterogeneity.

During cryopreservation, cells are typically cooled to cryogenic temperatures in the presence of cryoprotective agents (CPAs, e.g. glycerol, dimethyl sulfoxide, etc.) that limit the amount of damage caused by ice crystallization. Two traditional approaches to cryopreservation include slow freezing and vitrification. In slow freezing, CPAs (~1–2 M) are added to the cells followed by slow cooling in either a controlled rate freezer or specialized alcohol-filled freezing containers such as the Thermo Scientific^™^ Mr. Frosty^™^ Freezing Container. The appropriate cooling rate and concentration, incubation period and identity of CPA that confers protection during slow-freezing differs based upon the respective biophysical and biological characteristics unique to cell identity.[17, 18] Consequently, each cell type requires optimization of the cryopreservation protocol. Unlike slow cooling, vitrification is a method of “ice-free” cryopreservation where high concentrations of CPAs (~4–8 M) are added to cells followed by rapid cooling through the glass transition temperature to achieve glass formation. As this high concentration of CPA is toxic to most cell types, protocols for loading and unloading CPAs must be carefully developed to avoid cell mortality.[19–21] Because both slow cooling and vitrification require optimization for each cell line, CTC heterogeneity is a significant challenge to the successful development of standardized cryopreservation protocols. Further, CTC rarity imposes technical challenges as there is not sufficient biological material to optimize an appropriate cryopreservation protocol.

To address the need for standardized cryopreservation methods for cell lines, Heo *et al*. developed an ultra-fast vitrification protocol to enable cryogenic storage of a range of culture adapted and primary cells.[22] In ultra-fast vitrification cells are loaded into narrow (200 μm diameter) fused silica microcapillaries then plunged into liquid nitrogen to achieve a cooling rate of ~4000 K/s. Because cooling is so rapid, vitrification is achieved using substantially lower CPA concentrations (~1–2 M) compared to conventional approaches (~4–8 M). Due to the reduced concentration of CPA and absence of ice, ultra-fast vitrification is likely to be more broadly applicable to allow the long-term cryogenic storage of heterogeneous cell types, such as CTCs. Here, we have adapted ultra-fast vitrification to develop a standardized protocol to cryopreserve patient-derived CTCs. This method was validated using five CTC cell lines grown from metastatic breast cancer patients. Upon thawing, each CTC cell line exhibited high viability and normal growth in cell culture. EpCAM expression was also retained. Further evaluation of the BRx142 cell line by qPCR confirmed that common breast cancer biomarkers tested were unaffected by vitrification and RNA integrity was maintained.

## Materials and methods

### CTC cell lines and culture maintenance

Culture-adapted primary CTCs were obtained from peripheral blood from women with luminal B metastatic breast cancer. CTC isolation was performed using the CTC-iChip.[1, 16, 23] Culture-adapted CTCs were maintained in 6-well ultra-low adhesion plates (Corning CLS3471). Culture media and supplements were obtained from Life Technologies and consisted of RPMI 1640 medium (485 mL), EGF (10 μg), bFGF (10 μg), B27 (10 mL) and 100x Antibiotic-Antimycotic (5 mL). CTCs were maintained in a 5% CO_2_/5% O_2_/90% N_2_ incubator and subculture was performed after cells reached a density of ~500k cells/well. Cells were subcultured at a 1:3 ratio.

### Microcapillary preparation

Fused silica microcapillaries were obtained from Postnova Analytics Inc (UT, USA) with an inner diameter of 200 μm (item number Z-FSS-200280). Microcapillaries were cut into 7.5 cm lengths and the polyimide coating removed using the solvent N-methylpyrrolidone (NMP) then washed exhaustively with water to remove residual solvent. After drying overnight, tygon tubing (1.5 cm length) was placed on the end of the microcapillary. Cells were loaded by capillary action and expelled using a blunt needle attached to the tygon tubing.

### CPA loading and vitrification of cells

Cell-culture adapted CTCs were suspended in PBS and concentrated to approximately 10 million cells/mL by centrifugation for 3 min at 269xg using a swing bucket rotor. A 2μL aliquot was then added to a 1.5 mL centrifuge tube and diluted with 2 μL CPA stock consisting of 3 M Propanediol (PROH) and 0.5 M Trehalose. The sample was gently mixed with a pipette and incubated at room temperature for 1 minute followed by further addition of 6 μL CPA stock to achieve a final concentration of 2.4 M PROH and 0.4 M trehalose. After gently mixing the solution, cells were immediately loaded into microcapillaries and plunged into liquid nitrogen after a further 1 minute incubation (2 minute total CPA exposure). Cells were maintained in liquid nitrogen between 5 minutes and 24 hours. Using the microcapillaries, vitrification occurs in <1 second based on a ~4000 K/s cooling rate.[22] Thawing was achieved by plunging the microcapillary containing vitrified cells into a 37°C water bath for ~30 seconds. The cells were then expelled into a 1.5 mL centrifuge tube and diluted with an equivalent volume of PBS. Following a 2 minute incubation, cells were diluted to 100 μL. After gently mixing the cells, an aliquot was stained with Calcein AM and propidium iodide and imaged with a 20x objective using a Life Technologies EVOS FL microscope to determine viability. Percent viability was then normalized to the fresh, unfrozen control.

### Cell growth rate assays

Following vitrification using the described methods, thawed cells were then added to three separate 96-well clear bottom luminescence plate at a concentration of 3,000 viable cells/well. Control wells consisting of fresh, untreated cells were plated in a similar manner. All wells were filled to a final volume of 100 μL with CTC culture media. Cell growth was monitored using the CellTiterGlo assay following the manufacturer’s protocol. After 24 h incubation to allow cell recovery, the CellTiterGlo assay was performed to determine the baseline cell populations. Subsequent measurements were then taken on days three and five for BRx42, BRx50, BRx82 and BRx142 and normalized to the baseline reading to calculate percent growth. For BRx68, measurements were only taken after seven days of growth due to the slow doubling rate. This procedure was repeated at least three times for each cell line. Population doubling time was calculated from cell growth assays for each control. Data were analyzed using GraphPad Prism 6.

### EpCAM quantification using flow cytometry

CTCs were vitrified and thawed using the described protocol. CTCs were resuspended in 200 μL buffer solution (0.3% BSA and 10 mM HEPES in RPMI) immediately after removal of CPA. CTCs were stained with 1:285 PE-EpCAM (Cell Signaling Technologies), 1:500 CellEvent Caspase-3/7 Green (Life Technologies), and 12.5 μM Calcein Blue (Life Technologies) and stored in the dark for 20 minutes followed by treatment with 1:5000 DRAQ5 (Cell Signaling Technologies) for 15 minutes. Data analysis was performed using the IDEAS software package. Focused, single events were gated from the cell population resulting in >800 events for each vitrified sample. Gates were applied to determine EpCAM intensity and Calcein positivity. A fresh, unfrozen control was stained and analyzed for each CTC cell line immediately prior to vitrification for comparison.

### CTC RNA integrity and molecular expression

BRx142 CTCs were vitrified and stored for 24 h followed by thawing using the described protocol. Following CPA dilution, cell volume was adjusted to 20 μL. 5 μL of this cell suspension was stained with Calcein AM and propidium iodide to determine viability. Of the remaining cells, an aliquot containing 2,000 viable cells was transferred to a fresh 1.5 mL centrifuge tube, spun down (300 × g, 5 min), and resuspended in 200 μL of RNAlater. The sample was then flash frozen in liquid nitrogen and transferred to a -80°C freezer. This procedure was repeated seven times. Fresh, unfrozen controls were prepared in parallel (n = 6) in an identical manner. Total RNA was extracted using the Qiagen RNeasy Plus Micro Kit, as per manufacturer’s directions. RNA concentration was determined using a NanoDrop UV-VIS instrument, and sample purity was confirmed using absorbance at 260/280 nm. RNA quality was determined using the Bioanalyzer (Agilent Technologies), as per standard protocols, and are expressed as RNA Integrity Numbers (RIN). All data were analyzed using GraphPad Prism 6.

## Results

### Cryopreservation of heterogeneous CTC cell lines using a universal vitrification protocol

**Fig 1:**
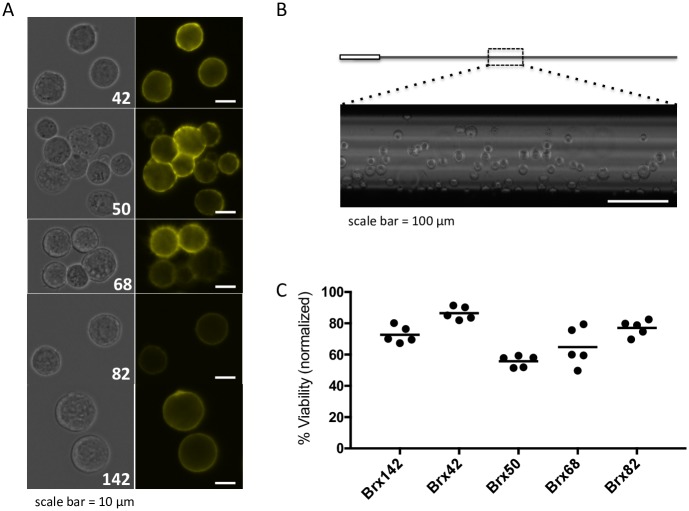
Vitrification of patient-derived CTCs. Five patient-derived CTC cell lines, BRx42, 50, 68, 82 and 142, were selected for this study. (A) The selected CTC cell lines demonstrate variation in size, growth as singles or clusters, and EpCAM intensity. (B) The microcapillaries used in this study are 200 μm in diameter and hold approximately 2 μL cell suspension. Insert shows capillary loaded with BRx42 cells (imaged with a 40x objective). (C) Each CTC cell line was vitrified using our standardized procedure. Excellent viability was observed after thawing where 55.7–86.4% of CTCs were viable.

As CTCs are extremely rare and challenging to isolate from whole blood, CTC cell lines were selected to serve as surrogates for this study. Five unique, culture-adapted CTC cell lines obtained from women with metastatic breast cancer were selected.[16] Fig 1A and Table 1 demonstrate heterogeneity between the CTC lines including differences in cell volume, EpCAM intensity, doubling rate, and growth as singles or clusters. As different cell lines typically require unique cryopreservation protocols, cellular heterogeneity is a significant hurdle for CTC biobanking. Here, the protocol reported by Heo, *et al*. was optimized for CTCs using the aforementioned CTC lines. CTCs were treated with 2.4 M PROH and 0.4 M Trehalose CPA solution and loaded into microcapillaries (200 μm diameter) then plunged into liquid nitrogen (Fig 1B). After thawing, cell viability was quantified and found to range from 55.7–86.4% (BRx42: 86.4 ± 4.1%; BRx50: 55.7 ± 3.7%; BRx68: 64.8 ± 12.3%; BRx82: 77.1 ± 5.0%; BRx142: 72.7 ± 5.4%; mean ± standard deviation, n = 5). BRx142 cells were further analyzed to determine yields. CPA-treated cells were loaded into microcapillaries at a concentration of 9–2,350 CTCs/microcapillary. After thawing samples, CTC recovery was quantified and found to be >85% for each cell concentration examined (S1 Fig).

**Table 1:** Population doubling time and volume of culture adapted CTC cell lines.

CTC Line	Doubling Rate (hours)	Cell Volume (fL)*
BRx42	71.8	2991 ± 978
BRx50	99.6	2905 ± 1213
BRx68	211.3	3488 ± 1354
BRx82	101.2	3597 ± 984
BRx142	59.7	5143 ± 1317

*geometric mean ± mean absolute deviation

### CTC EpCAM expression is not reduced by vitrification

**Fig 2:**
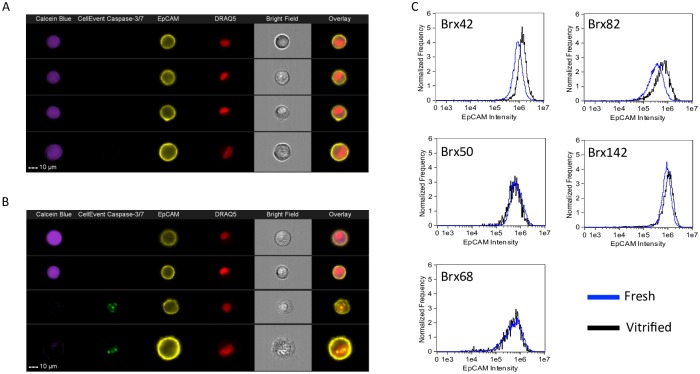
Analysis of EpCAM expression following vitrification. (A) Fresh and (B) vitrified CTCs were evaluated using imaging flow cytometry to quantify EpCAM expression (BRx42 cells shown). (C) Histograms of the relative EpCAM intensity shows no deterioration of signal among the CTC cell lines.

Critical to CTC enumeration is the presentation of surface markers to enable differentiation from normal circulating blood cells. Epithelial cell adhesion molecule (EpCAM/CD326) is a transmembrane glycoprotein expressed in epithelial-derived tumors and is commonly used as a marker for CTC enrichment.[24] Here, CTC EpCAM expression was quantified to ensure that vitrification does not interfere with EpCAM expression. CTCs were stained with PE-EpCAM immediately after thawing and analyzed by flow cytometry for comparison to fresh, unfrozen controls. CTCs were also stained with Calcein Blue viability marker, CellEvent Caspase-3/7 apoptosis marker and DRAQ5 nuclear marker. Fig 2A shows the baseline EpCAM expression levels for fresh BRx42 CTCs compared to vitrified CTCs (Fig 2B). Both live/nonapoptotic (calcein^+^/CellEvent^-^) and dead/apoptotic cells (calcein^-^/CellEvent^+^) appear to maintain robust EpCAM expression. Further quantification of total EpCAM intensity reveals that the signal intensity is maintained among the vitrified CTCs compared to the fresh controls in each of the five CTC lines examined (Fig 2C). These results suggest that CTC cryopreservation by ultra-fast vitrification does not impair the presentation of EpCAM and therefore does not interfere with CTC identification in assays that rely on this tumor associated biomarker.

### Cell growth rates are maintained following CTC vitrification

**Fig 3:**
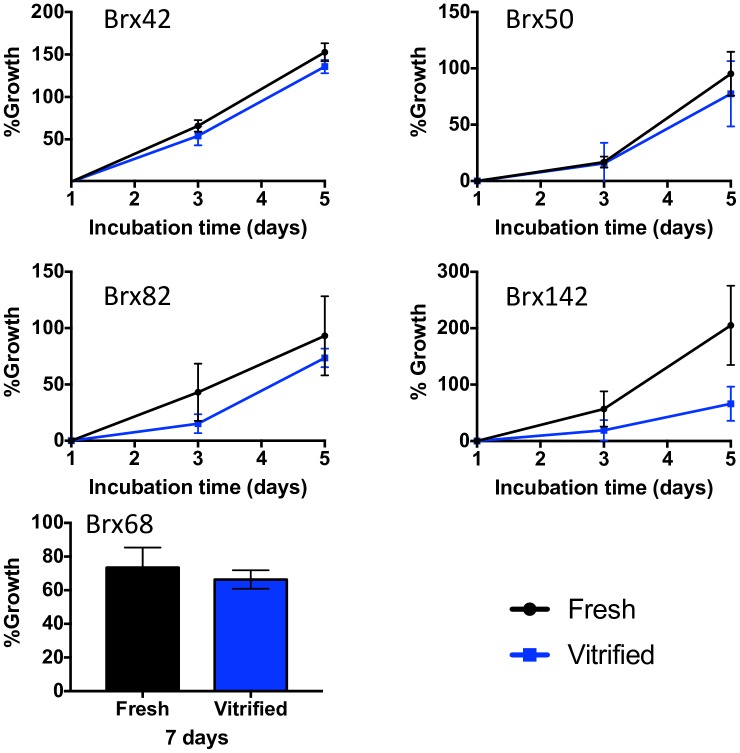
Cell culture of vitrified CTCs. CTC growth in culture was characterized for fresh and vitrified cells. Each cell line was monitored on Day 1, 3 and 5, with the exception of BRx68 which was measured on Day 1 and 8 only due to the slow doubling rate.

The viability data reveals excellent survival rates among the CTC lines immediately after thawing (55.7–86.4%). As CTC cell culture is an increasing area of research interest, the growth rate of vitrified CTCs was quantified for comparison to fresh, unfrozen CTCs to determine whether cells were negatively impacted from cryogenic storage. BRx CTC lines were vitrified, plated into 96-well plates, and growth was quantified using the CellTiterGlo assay at 1, 3 and 5 days after thawing, with the exception of BRx68 which was monitored at 1 and 8 days only due to slow doubling rate. We observed excellent growth rates for BRx42, BRx50 and BRx82 where no significant deviation from the control was observed at any time point (Fig 3). Likewise, after 7 days of incubation, BRx68 growth rates for vitrified CTCs was comparable to that of fresh controls. In the case of BRx142, a significant delay in cell growth was observed (p<0.0001, 2-way ANOVA with Bonferroni correction for multiple comparisons), though the cells did continue to expand *in vitro* during the measured time period.

### RNA Integrity Numbers and molecular expression is stable following CTC vitrification

**Fig 4:**
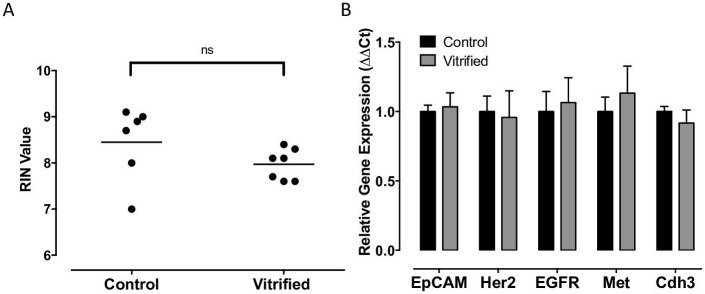
Molecular analysis of vitrified CTCs. (A) RINs were obtained for fresh (control) and vitrified BRx142 cells to determine whether RNA integrity was compromised. No significant loss in RNA integrity was observed following cryogenic storage (control n = 6, vitrified n = 7). (B) Gene expression signatures were further evaluated for the BRx142 cells using qPCR to detect changes in common breast cancer biomarkers. No significant loss was observed.

CTCs are a rich source of molecular information that may provide clinical information regarding the status of the primary tumor.[6–10] Therefore, it was important to determine whether RNA integrity and relative gene expression of common tumor specific transcripts were impacted by vitrification. Fig 4A shows that vitrified BRx142 CTCs exhibit no significant deviation in RIN value compared to fresh, unfrozen controls. Based upon the positive results, qPCR was performed to determine whether vitrification had an effect on relative gene expression for the BRx142 CTC cell line. We chose a panel of common breast cancer biomarkers, including EpCAM, Her2, EGFR, Met and Cdh3. Among the selected biomarkers, no significant deviation from control levels was observed (Fig 4B).

## Discussion

Here, we evaluated an ultra-fast vitrification approach as a standardized cryopreservation protocol for patient-derived CTCs. The approach uses silica microcapillaries to achieve extremely fast cooling rates in order to vitrify with low CPA concentrations. This method was validated using five culture-adapted CTC cell lines obtained from metastatic breast cancer patients. Upon thawing, each CTC line exhibited high viability and normal growth in cell culture. EpCAM expression was retained and further evaluation of the BRx142 cell line by qPCR confirmed that common breast cancer biomarkers tested were unaltered by vitrification.

Microfluidic technologies have enabled the isolation of extremely rare CTCs from peripheral blood.[1–5] These CTCs represent a rich source of molecular information that may provide clinical information regarding the status of the primary tumor, disease prognosis and provide opportunities to improve cancer management.[6–10] In particular, serial measurements of CTC genotype during gefitinib therapy showed that cells evolved throughout treatment and enabled the detection of drug-resistance mutations.[7] E*x vivo* CTC cultures also provide an opportunity to test individual drug susceptibility.[16] To support these clinical and research endeavors, methods for CTC cryopreservation are needed to enable storage for these fragile cells.

Recently, Heo *et al*. described a universal method to vitrify cells using ultra-fast cooling in silica microcapillaries.[22, 25] This method was validated against a range of cells including cancer cell lines, patient derived tumor cells, a fibroblast cell line and primary rat hepatocytes. The success of this method was attributed to the high cooling rates achieved by microcapillaries. As the concentration of CPA required for vitrification is inversely related to cooling rate, conventional vitrification typically requires very high CPA concentrations (~4–8 M), which are toxic to most cells and thus requires carefully optimized protocols depending upon the sensitivity to the CPA.[19–21] The CPA concentration utilized by ultra-fast vitrification (~1–2 M) is less toxic yet still forms a vitreous state and is therefore more universally applicable for the vitrification of heterogeneous cell suspensions. Here, we evaluated this approach for the vitrification of CTCs. Using CTC cell lines obtained from metastatic breast cancer patients, we observed high post-thaw viability (55.7–86.4%). Titrations also reveal that >85% CTC recovery may be achieved following vitrification of CTCs at cell density of 9–2,350 CTCs/microcapillary (S1 Fig). Vitrification did not prevent expansion in cell culture. Importantly, EpCAM expression was conserved regardless of viability post-thaw (Fig 2). Retention of surface markers such as EpCAM is critical for CTC enumeration. Vitrification also had no impact on the transcripts examined in this study.

A standardized cryopreservation method for CTCs would address the needs for both temporary and long-term storage methods. Temporary methods for CTC storage would ease experimental timelines as isolation and subsequent analysis (i.e. enumeration or molecular characterization) are time intensive processes. The development of a standardized, universally applicable cryopreservation procedure for CTCs would ease these restrictive timelines, allowing more efficient batch processing of isolates at a later time. This could be particularly useful for clinical trials where serial specimens could be stored for later analysis. Banking a portion of the CTCs from each measurement would enable later characterization of material as new molecular methods become available. A challenge to the development of a standardized procedure is the heterogeneity of CTCs as cells require uniquely optimized cryopreservation protocols based on their specific biophysical and biochemical characteristics.[17, 18] Many forms of cellular heterogeneity among CTCs have been described including biophysical features such as size and presence of clusters, genomic features, expression of surface markers and expression of epithelial or mesenchymal markers.[26] This inter- and intra-patient cellular heterogeneity combined with CTC rarity represents a technical barrier to the optimization of cryopreservation methods for unique isolates, necessitating the development of a standardized protocol.

Here, we have described a general method to vitrify CTCs and validated this method using five patient-derived CTC cell lines. This is a valuable approach that accomplishes both temporary storage to ease experimental timelines and enable batch processing and for longer storage as needed in biobanking. Future work will aim to validate this approach in CTCs isolated from peripheral blood of patients with various forms of metastatic cancer.

## Supporting information

S1 Fig Optimization of CTC recovery. BRx142 CTCs were treated with CPA and loaded into microcapillaries at the indicated concentration (microcapillary volume capacity is 2 μL). Tygon tubing was placed on both ends of the microcapillary and sealed with a heat sealer. Microcapillaries were then vitrified and thawed. CTCs were then washed out of the microcapillaries with 8 μL PBS and counted with a hemacytometer to determine the percent recovery relative to the control (n = 3 for each condition). For the control, 2 μL of CTCs were transferred directly from the stock and mixed with 8 μL of PBS then counted on a hemacytometer in order to determine the percent recovery of CTCs in the vitrified samples.
(PDF)

## References

[1] Karabacak NM, Spuhler PS, Fachin F, Lim EJ, Pai V, Ozkumur E, et al Microfluidic, marker-free isolation of circulating tumor cells from blood samples. Nat Protoc. 2014;9(3):694–710. doi: 10.1038/nprot.2014.044 24577360 10.1038/nprot.2014.044 PMC4179254

[2] Stott SL, Hsu CH, Tsukrov DI, Yu M, Miyamoto DT, Waltman BA, et al Isolation of circulating tumor cells using a microvortex-generating herringbone-chip. Proc Natl Acad Sci U S A. 2010;107(43):18392–7. doi: 10.1073/pnas.1012539107 20930119 10.1073/pnas.1012539107 PMC2972993

[3] Warkiani ME, Khoo BL, Wu L, Tay AK, Bhagat AA, Han J, et al Ultra-fast, label-free isolation of circulating tumor cells from blood using spiral microfluidics. Nat Protoc. 2016;11(1):134–48. doi: 10.1038/nprot.2016.003 26678083 10.1038/nprot.2016.003

[4] Hou HW, Warkiani ME, Khoo BL, Li ZR, Soo RA, Tan DS, et al Isolation and retrieval of circulating tumor cells using centrifugal forces. Sci Rep. 2013;3:1259 doi: 10.1038/srep01259 23405273 10.1038/srep01259 PMC3569917

[5] Gleghorn JP, Pratt ED, Denning D, Liu H, Bander NH, Tagawa ST, et al Capture of circulating tumor cells from whole blood of prostate cancer patients using geometrically enhanced differential immunocapture (GEDI) and a prostate-specific antibody. Lab Chip. 2010;10(1):27–9. doi: 10.1039/b917959c 20024046 10.1039/b917959c PMC3031459

[6] Punnoose EA, Atwal SK, Spoerke JM, Savage H, Pandita A, Yeh RF, et al Molecular biomarker analyses using circulating tumor cells. PLoS One. 2010;5(9):e12517 doi: 10.1371/journal.pone.0012517 20838621 10.1371/journal.pone.0012517 PMC2935889

[7] Maheswaran S, Sequist LV, Nagrath S, Ulkus L, Brannigan B, Collura CV, et al Detection of mutations in EGFR in circulating lung-cancer cells. N Engl J Med. 2008;359(4):366–77. doi: 10.1056/NEJMoa0800668 18596266 10.1056/NEJMoa0800668 PMC3551471

[8] Barbazán J, Alonso-Alconada L, Muinelo-Romay L, Vieito M, Abalo A, Alonso-Nocelo M, et al Molecular characterization of circulating tumor cells in human metastatic colorectal cancer. PLoS One. 2012;7(7):e40476 doi: 10.1371/journal.pone.0040476 22811761 10.1371/journal.pone.0040476 PMC3397799

[9] Harouaka R, Kang Z, Zheng SY, Cao L. Circulating tumor cells: advances in isolation and analysis, and challenges for clinical applications. Pharmacol Ther. 2014;141(2):209–21. doi: 10.1016/j.pharmthera.2013.10.004 24134902 10.1016/j.pharmthera.2013.10.004 PMC3947247

[10] Hou JM, Krebs MG, Lancashire L, Sloane R, Backen A, Swain RK, et al Clinical significance and molecular characteristics of circulating tumor cells and circulating tumor microemboli in patients with small-cell lung cancer. J Clin Oncol. 2012;30(5):525–32. doi: 10.1200/JCO.2010.33.3716 22253462 10.1200/JCO.2010.33.3716

[11] Baker M. Biorepositories: Building better biobanks. Nature. 2012;486(7401):141–6. doi: 10.1038/486141a 22678297 10.1038/486141a

[12] Liu A, Pollard K. Biobanking for Personalized Medicine. Adv Exp Med Biol. 2015;864:55–68. doi: 10.1007/978-3-319-20579-3_5 26420613 10.1007/978-3-319-20579-3_5

[13] Baccelli I, Schneeweiss A, Riethdorf S, Stenzinger A, Schillert A, Vogel V, et al Identification of a population of blood circulating tumor cells from breast cancer patients that initiates metastasis in a xenograft assay. Nat Biotechnol. 2013;31(6):539–44. doi: 10.1038/nbt.2576 23609047 10.1038/nbt.2576

[14] Hodgkinson CL, Morrow CJ, Li Y, Metcalf RL, Rothwell DG, Trapani F, et al Tumorigenicity and genetic profiling of circulating tumor cells in small-cell lung cancer. Nat Med. 2014;20(8):897–903. doi: 10.1038/nm.3600 24880617 10.1038/nm.3600

[15] Girotti MR, Gremel G, Lee R, Galvani E, Rothwell D, Viros A, et al Application of Sequencing, Liquid Biopsies, and Patient-Derived Xenografts for Personalized Medicine in Melanoma. Cancer Discov. 2016;6(3):286–99. doi: 10.1158/2159-8290.CD-15-1336 26715644 10.1158/2159-8290.CD-15-1336

[16] Yu M, Bardia A, Aceto N, Bersani F, Madden MW, Donaldson MC, et al Ex vivo culture of circulating breast tumor cells for individualized testing of drug susceptibility. Science. 2014;345(6193):216–20. doi: 10.1126/science.1253533 25013076 10.1126/science.1253533 PMC4358808

[17] Mazur P. Cryobiology: the freezing of biological systems. Science. 1970;168(3934):939–49. 5462399 10.1126/science.168.3934.939

[18] Stacey GN, Masters JR. Cryopreservation and banking of mammalian cell lines. Nat Protoc. 2008;3(12):1981–9. doi: 10.1038/nprot.2008.190 19180080 10.1038/nprot.2008.190

[19] Hunt CJ, Pegg DE, Armitage SE. Optimising cryopreservation protocols for haematopoietic progenitor cells: a methodological approach for umbilical cord blood. Cryo Letters. 2006;27(2):73–86. 16794739

[20] Fahy GM, Wowk B, Wu J, Paynter S. Improved vitrification solutions based on the predictability of vitrification solution toxicity. Cryobiology. 2004;48(1):22–35. doi: 10.1016/j.cryobiol.2003.11.004 14969679 10.1016/j.cryobiol.2003.11.004

[21] Karlsson JO, Toner M. Long-term storage of tissues by cryopreservation: critical issues. Biomaterials. 1996;17(3):243–56. 8745321 10.1016/0142-9612(96)85562-1

[22] Heo YS, Nagrath S, Moore AL, Zeinali M, Irimia D, Stott SL, et al "Universal" vitrification of cells by ultra-fast cooling. Technology (Singap World Sci). 2015;3(1):64–71. 25914896 10.1142/S2339547815500053 PMC4404302

[23] Ozkumur E, Shah A, M., Ciciliano JC, Emmink BL, Miyamoto DT, Brachtel E, et al Inertial Focusing for Tumor Antigen-Dependent and -Independent Sorting of Rare Circulating Tumor Cells. Science Translational Medicine. 2013;5(179):179ra47 doi: 10.1126/scitranslmed.3005616 23552373 10.1126/scitranslmed.3005616 PMC3760275

[24] Allard WJ, Matera J, Miller MC, Repollet M, Connelly MC, Rao C, et al Tumor cells circulate in the peripheral blood of all major carcinomas but not in healthy subjects or patients with nonmalignant diseases. Clin Cancer Res. 2004;10(20):6897–904. doi: 10.1158/1078-0432.CCR-04-0378 15501967 10.1158/1078-0432.CCR-04-0378

[25] Risco R, Elmoazzen H, Doughty M, He X, Toner M. Thermal performance of quartz capillaries for vitrification. Cryobiology. 2007;55(3):222–9. doi: 10.1016/j.cryobiol.2007.08.006 17919532 10.1016/j.cryobiol.2007.08.006

[26] Bulfoni M, Turetta M, Del Ben F, Di Loreto C, Beltrami AP, Cesselli D. Dissecting the Heterogeneity of Circulating Tumor Cells in Metastatic Breast Cancer: Going Far Beyond the Needle in the Haystack. Int J Mol Sci. 2016;17(10). 10.3390/ijms17101775 PMC5085799 27783057

