# Supplementary material for: Ultra-fast vitrification of patient-derived circulating tumor cell lines
Source: PLoS One. 2018 Feb 23;13(2):e0192734. doi: 10.1371/journal.pone.0192734 (PMC5825040; doi:10.1371/journal.pone.0192734)
Supplement: S1 Fig — BRx142 CTCs were treated with CPA and loaded into microcapillaries at the indicated concentration (microcapillary volume capacity is 2 μL). Tygon tubing was placed on both ends of the microcapillary and sealed with a heat sealer. Microcapillaries were then vitrified and thawed. CTCs were then washed out of the microcapillaries with 8 μL PBS and counted with a hemacytometer to determine the percent recovery relative to the control (n = 3 for each condition). For the control, 2 μL of CTCs were transferred directly from the stock and mixed with 8 μL of PBS then counted on a hemacytometer in order to determine the percent recovery of CTCs in the vitrified samples. (PDF) [file pone.0192734.s001.pdf]

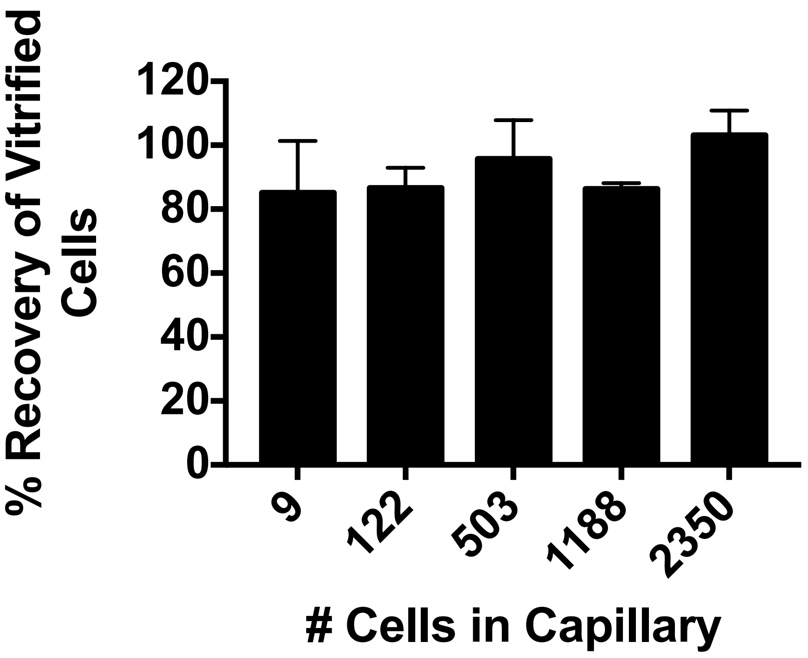

Supplemental Figure 1. Optimization of CTC recovery. BRx142 CTCs were treated with CPA and loaded into capillaries at the indicated concentration (capillary volume capacity is 2 μL). Tygon tubing was placed on both ends of the capillary and sealed with a heat sealer. Capillaries were then vitrified and thawed. CTCs were then washed out of the capillaries with 8 μL PBS and counted with a hemacytometer to determine the percent recovery relative to the control (n=3 for each condition). For the control, 2 μL of CTCs were transferred directly from the stock and mixed with 8 μL of PBS then counted on a hemacytometer in order to determine the percent recovery of CTCs in the vitrified samples.
